# Summary of the National Advisory Committee on Immunization (NACI) rapid response: Updated guidance on the use of Imvamune® for the prevention of mpox

**DOI:** 10.14745/ccdr.v52i04a02

**Published:** 2026-04-30

**Authors:** Joshua Montroy, Marina Salvadori, Nicole Forbes, Kristin Klein

**Affiliations:** 1 Centre for Immunization Surveillance and Programs, Public Health Agency of Canada, Ottawa, ON; 2 Department of Pediatrics, McGill University, Montréal, QC; 3 Department of Medicine, University of Alberta, Edmonton, AB

**Keywords:** National Advisory Committee on Immunization, NACI, mpox, guidance, Imvamune

## Abstract

**Background:**

The National Advisory Committee on Immunization (NACI) previously released guidance in May of 2024 regarding the use of Imvamune® for the prevention of mpox in the context of a routine immunization program. Since the release of this guidance, the emergence of a novel subclade of mpox (clade Ib) has led to a shifting epidemiological landscape and several international jurisdictions have, therefore, revised their Imvamune guidance.

**Objective:**

To summarize the updated NACI guidance on the use of Imvamune for the prevention of mpox.

**Methods:**

NACI reviewed data on the current epidemiology of mpox, both in Canada and internationally, current evidence on Imvamune vaccination coverage in Canada, and published scientific literature regarding the effectiveness, immunogenicity and safety of Imvamune. NACI has also considered additional factors including ethics, equity, feasibility and acceptability. Guidance on the use of Imvamune in the context of international travel was developed in collaboration with the Canadian Committee to Advise on Tropical Medicine and Travel.

**Results:**

NACI concluded that available evidence supports the limited expansion of groups considered at high risk of mpox.

**Conclusion:**

NACI now recommends that in addition to previously identified groups at high risk of mpox, certain travellers to areas with ongoing community transmission of mpox virus clade I should receive Imvamune. Healthcare workers travelling internationally to support mpox outbreaks should also receive Imvamune, and domestic healthcare workers with a high risk of frequent exposure may consider vaccination with Imvamune on an individual basis.

## Introduction

Mpox is caused by the monkeypox virus (MPXV), a mammalian *Orthopoxvirus,* and is transmitted either by zoonotic (animal-to-human) contact or by person-to-person contact with bodily fluids, lesions on the skin or mucosal surfaces (e.g., mouth, throat, anogenital region) ((1)). The MPXV is subclassified into two distinct clades (clades I and II) and further classified into subclades (subclades Ia and Ib, and subclades IIa and IIb), with clade IIb causing the 2022 global outbreak. Mpox is typically a mild and self-limited disease; however, it can lead to severe disease in specific populations, including young children ((2)), individuals who are pregnant ((3)) and individuals who are immunocompromised ((4)).

In August 2024, the World Health Organization (WHO) declared mpox a public health emergency of international concern for the second time ((5)), following a rapid increase in cases linked to a novel subclade (clade Ib) in the Democratic Republic of the Congo. Between January 1, 2024, and January 26, 2025, a total of 19,837 confirmed mpox cases and 93 deaths have been reported in Africa ((6)). The number of suspected mpox cases and mpox-related deaths was considerably higher. Travel-related cases of mpox clade Ib have since been reported in numerous countries outside of Africa, including in Canada.

As of September 26, 2025, ten Canadian provinces and territories have publicly reported 2,236 cases of mpox, with the majority of cases occurring in Ontario (52.2%), Québec (27.6%) and British Columbia (16.4%) ((7)). Two cases of mpox clade I (clade Ib) have been reported in Canada (as of September 26, 2025), both travel-related. In Canada, gay, bisexual and other men who have sex with men (GBMSM) continue to experience the largest disease burden, with over 95% of mpox cases reported. The majority of mpox cases observed in Canada occurred during the initial outbreak in 2022 (approximately 66%, n=1,477 cases), with most cases having been reported in Ontario, Québec and British Columbia. There have been 677 mpox cases reported in Canada since the start of 2024 (as of September 26, 2025). Severe disease remains relatively rare, with 53 hospitalizations (seven since the start of 2024), three intensive care unit admissions (none since the start of 2024) and no deaths recorded to date in Canada.

The National Advisory Committee on Immunization (NACI) previously provided interim guidance on the use of Imvamune (Modified vaccinia Ankara-Bavarian Nordic [MVA-BN]) on May 24, 2024 ((8)), in the context of a routine immunization program. Given the evolving global mpox outbreak and emerging evidence on vaccine effectiveness, NACI issued updated guidance on the use of Imvamune for the prevention of mpox in May of 2025, and this updated guidance is summarized below.

## Methods

For this rapid response, NACI reviewed key questions as proposed for the NACI mpox Working Group regarding the use of Imvamune, considering reports of ongoing mpox clade I outbreaks globally. Data on the current epidemiology of mpox, both in Canada and internationally, current evidence on Imvamune vaccination coverage in Canada, and published scientific literature regarding the effectiveness, immunogenicity and safety of Imvamune were reviewed. Knowledge synthesis was performed by the NACI Secretariat and supervised by the NACI mpox Working Group. Following critical appraisal of individual studies, summary tables with risk of bias ratings informed by Cochrane 2.0 or ROBINS-I, as appropriate, were prepared. Stakeholders that provided feedback included a Canadian group representing GBMSM communities, the National Emergency Strategic Stockpile, the Canadian Immunization Committee and the Committee to Advise on Tropical Medicine and Travel (collaborated on guidance related to international travel). NACI reviewed the available evidence and approved the updated recommendations on February 18, 2025. Further information on NACI’s evidence-based methodology is available online.

## Results

### Evolving mpox epidemiology

Evidence directly comparing the severity of MPXV clades and subclades, including any data stratified by age, sex or other risk factors (i.e., immunocompromised status) are still emerging ((9–13)). In non-endemic areas, clade IIb transmission continues to largely occur in high-contact sexual networks, primarily the GBMSM community and, therefore, the majority of cases reported have been amongst adult males. Unlike clade IIb, the strain responsible for the 2022 global outbreak, there is considerable transmission of both MPXV clade Ia and Ib outside of GBMSM sexual networks. In mpox clade Ia affected regions, there is a large disease burden in heterosexual males and females, as well as children ((9–14)). Of the two clade I subclades, clade Ia appears to be associated with greater disease severity and a higher mortality rate compared to clade Ib ((15–17)). Given the limitations of available data from MPXV endemic countries, it is also possible that observed differences between MPXV clades and subclades are driven by differences in demographic variables (including the populations affected, access to healthcare, nutrition, etc.) between infected individuals.

### Vaccine immunogenicity

Recent data have suggested that the antibody titres produced by Imvamune vaccination in mpox-naive individuals wane over time, usually within six to 12 months ((18–20)). The clinical significance of declining antibody levels over time remains unclear due to the lack of a defined correlate of protection for Imvamune. It is possible that there are non-antibody markers of protection, as the role of innate and cell-mediated immunity is not currently fully understood. In addition, the robustness of memory responses may potentially influence the disease outcome. The disparate time intervals observed between vaccination and breakthrough infection among fully vaccinated individuals supports this theory ((21)).

### Vaccine effectiveness against mpox infection and severe disease

Imvamune administered as a single dose or two-dose schedule for the prevention of mpox infection continues to demonstrate a high degree of effectiveness. A recent systematic review and meta-analysis ((22)) reported one-dose vaccine effectiveness was 76% (95% CI: 64%–88%) and two-dose vaccine effectiveness was 82% (95% CI: 72%–92%).

Data on the effectiveness of Imvamune against moderate to severe mpox infection (including hospitalization) is less robust, but encouraging. In studies providing estimates of effect, even a single dose of Imvamune was effective at reducing moderate to severe mpox infection (vaccine effectiveness: 82%; 95% CI: −50%–98%) ((23)) and at reducing the odds of hospitalization due to mpox (odds ratio [OR]: 0.27; 95% CI: 0.08–0.65) ((24)). Similarly, a two-dose schedule was also associated with a significant decrease in the odds of hospitalization due to mpox (OR: 0.20; 95% CI: 0.01–0.90 and OR: 0.20; 95% CI: 0.0–0.5) ((21,24)). Several additional studies have demonstrated the rarity of hospitalizations due to mpox in individuals who have been vaccinated with Imvamune®, even with a single dose ((25–28)). When breakthrough infection does occur, the severity of infection appears considerably reduced following vaccination with Imvamune, with fewer lesions, less mucosal involvement, and fewer systemic symptoms ((21,25,27)).

At the time of NACI guidance development, available vaccine effectiveness data comes from studies conducted in countries where mpox is non-endemic, where MPXV clade IIb is responsible for virtually all mpox cases and therefore the effectiveness of Imvamune against MPXV clade Ia and Ib is unclear at this time. Unpublished data from a Centers for Disease Control and Prevention study in the Democratic Republic of the Congo that began in 2017 and vaccinated approximately 1,600 healthcare workers with two doses of Imvamune, observed only one laboratory-confirmed infection, indicating significant effectiveness against mpox in an area of high clade I transmission ((29,30)). In addition, several challenge studies in non-human primates have demonstrated significant protection against MPXV clade I associated with MVA-BN vaccination ((31–34)). There is currently no evidence and no biological rationale to suggest a decrease in vaccine effectiveness against MPXV clade Ia or Ib.

### Vaccine safety

Available post-marketing safety surveillance data on Imvamune suggests that the vaccine is well-tolerated. The most common adverse events reported by adults following one and/or two doses were non-serious injection-site and systemic reactions, consistent with clinical trial findings. Generally, the second dose was slightly better tolerated than the first. No serious adverse events were reported after either dose, including no signals for increased risks of myocarditis/pericarditis following vaccination.

Data regarding the safety and tolerability of a third dose of Imvamune are limited to two small clinical trials. A randomized, phase II trial in adults living with HIV administered a booster dose of Imvamune to 31 participants, 12 weeks following the second dose ((35)). Compared to a group of participants receiving the standard two-dose regimen, those receiving a booster dose reported a higher incidence of solicited local adverse events; however, no serious adverse events or adverse events of special interest related to study vaccine were reported. Unpublished results from a phase II clinical trial in Germany ((36)) reported similar results, with no serious adverse events related to the vaccine being observed following a third dose two years after completion of the two-dose series (n=75 participants). One participant (1.3%) reported an unsolicited grade three or higher adverse event within 29 days of vaccination. A recent study of healthcare workers from the Democratic Republic of the Congo, who received a third dose five years after completion of the primary series, demonstrated elevated rates of local and systemic reactions following the administration of the third dose, compared to the first or second dose of the primary series. However, no severe adverse events were observed ((37)).

### Ethics, equity, feasibility and acceptability considerations

During the development of initial recommendations for Imvamune use for the prevention of mpox, Public Health Agency of Canada consulted with stakeholder groups representing impacted communities. Overall, GBMSM communities communicated positive attitudes towards mpox vaccination; however, since 2022, the majority of Imvamune recipients have only had their first dose. This could potentially be due to factors such as perceived lower risk of infection compared to the spring/summer of 2022 when case numbers were high across many Canadian urban centers, or perceived risk of adverse events following immunization after the first dose of Imvamune ((38–40)).

All Canadian provinces and territories continue to offer Imvamune to individuals considered at high risk of mpox; however, understanding potential barriers and challenges to vaccination from the end-user perspective is critical to improving vaccine uptake ((41)). It has been noted that identification of travel-associated risks (i.e., sex tourism) presents feasibility and equity challenges. Travel-associated risks are difficult to identify through public health clinics in some jurisdictions, and this impacts feasibility of operationalizing recommendations relating to travel risk. It has also been identified that vaccine programs administered in travel clinics based on travel risk assessment can incur individual costs, which can create inequities. Despite feasibility and potential equity challenges, NACI and Committee to Advise on Tropical Medicine and Travel considered it important to support vaccine access to travelers who may be at increased risk of mpox due to the evolving global situation. An additional barrier to access for those seeking vaccination includes the fact that due to the regulatory status of this vaccine in Canada, and because the main supply is held federally, Imvamune cannot be purchased by individuals on the private market and can only be accessed through provincial, territorial or federal programs for recommended populations.

## NACI recommendations

**Recommendation 1:** NACI continues to recommend that individuals at high risk of mpox should receive two doses of Imvamune administered at least 28 days (four weeks) apart. **(*Strong NACI recommendation*)**

• At this time individuals considered at high risk of mpox in Canada include the following:

o Men who have sex with men (MSM) who:

▪ Have more than one partner

▪ Are in a relationship where at least one of the partners has other sexual partners

▪ Have had a confirmed sexually transmitted infection acquired in the last year

▪ Have engaged in sexual contact in sex-on-premises venues

o Sexual partners of individuals who meet the criteria above

o Sex workers regardless of gender, sex assigned at birth or sexual orientation

o Staff or volunteers in sex-on-premises venues where workers may have contact with fomites potentially contaminated with mpox

o Individuals who anticipate experiencing any of the above scenarios, including during travel outside of Canada

Also at high risk are individuals who are travelling to an area with ongoing community transmission of MPXV clade I (see mpox: Advice for travellers for a list of countries meeting this criteria) and anticipate either of the following:

• Prolonged close contact (e.g., sharing accommodation), with people who reside in the area of active transmission

• Sexual contact with people who reside in, or spend extended time in, the area of active transmission

Note: NACI guidance related to international travel has been updated above, in collaboration with Committee to Advise on Tropical Medicine and Travel, to reflect current mpox epidemiology worldwide.

**Recommendation 2:** At this time, Imvamune is not routinely recommended for healthcare workers, with the exception of post-exposure vaccination. However, it may be considered on an individual basis, based on a high risk of frequent exposure (e.g., healthcare workers who work at clinics that are frequently involved in the diagnosis and management of mpox). **(*Discretionary NACI recommendation*)**

**Recommendation 3:** NACI recommends that healthcare workers who are travelling internationally to support mpox outbreaks should be vaccinated with Imvamune ahead of deployment. **(*Strong NACI recommendation*)**

**Recommendation 4A:** NACI continues to recommend that personnel who work in research laboratory settings and who are at high risk of occupational exposure to replicating orthopoxviruses that pose a risk to human health should receive two doses of Imvamune administered at least 28 days (four weeks) apart. **(*Strong NACI recommendation*)**

**Recommendation 4B:** NACI recommends that additional doses of Imvamune may be offered for personnel who work in a research laboratory setting and who remain at high risk of occupational exposure to replicating orthopoxviruses that pose a risk to human health, with a minimum interval of two years. **(*Discretionary NACI recommendation*)**

**Recommendation 5:** NACI continues to recommend the use of Imvamune as post-exposure vaccination (also known and referred to as post-exposure prophylaxis) to individuals who have had high risk exposure(s) to a probable or confirmed case of mpox, or within a setting where transmission is happening, if they have not received both doses of pre-exposure vaccination. **(*Strong NACI recommendation*)**

## Conclusion

Considering the evolving mpox epidemiology and emerging data regarding the vaccine effectiveness of Imvamune, NACI has provided updated guidance on the use of Imvamune for the prevention of mpox. Updates include the addition of travellers to areas of with ongoing community transmission of MPXV clade I circulation to the groups recommended to receive Imvamune, as well updated recommendations for healthcare workers. NACI recommendations on the use of Imvamune are based on available clinical evidence and current mpox epidemiology and may be re-evaluated if additional evidence emerges.
